# Supplementary material for: Unraveling the Diverse Profile of N-Acyl Homoserine Lactone Signals and Their Role in the Regulation of Biofilm Formation in Porphyra haitanensis-Associated Pseudoalteromonas galatheae
Source: Microorganisms. 2023 Sep 4;11(9):2228. doi: 10.3390/microorganisms11092228 (PMC10537045; doi:10.3390/microorganisms11092228)
Supplement: Supplementary file 1 [file microorganisms-11-02228-s001.zip › microorganisms-2551662-supplementary.pdf]

Unraveling the Diverse Profile of N-Acyl Homoserine Lactones Signals and their Role in the Regulation of Biofilm Formation in *Porphyra haitanensis* associated *Pseudoalteromonas galatheae*

Muhammad Aslam [1,2], Pengbing Pei [1], Peilin Ye [1], Tangcheng Li [1], Honghao Liang [1], Zezhi Zhang [1], Xiao Ke [1], Weizhou Chen [1] and Hong Du [1,3,*]

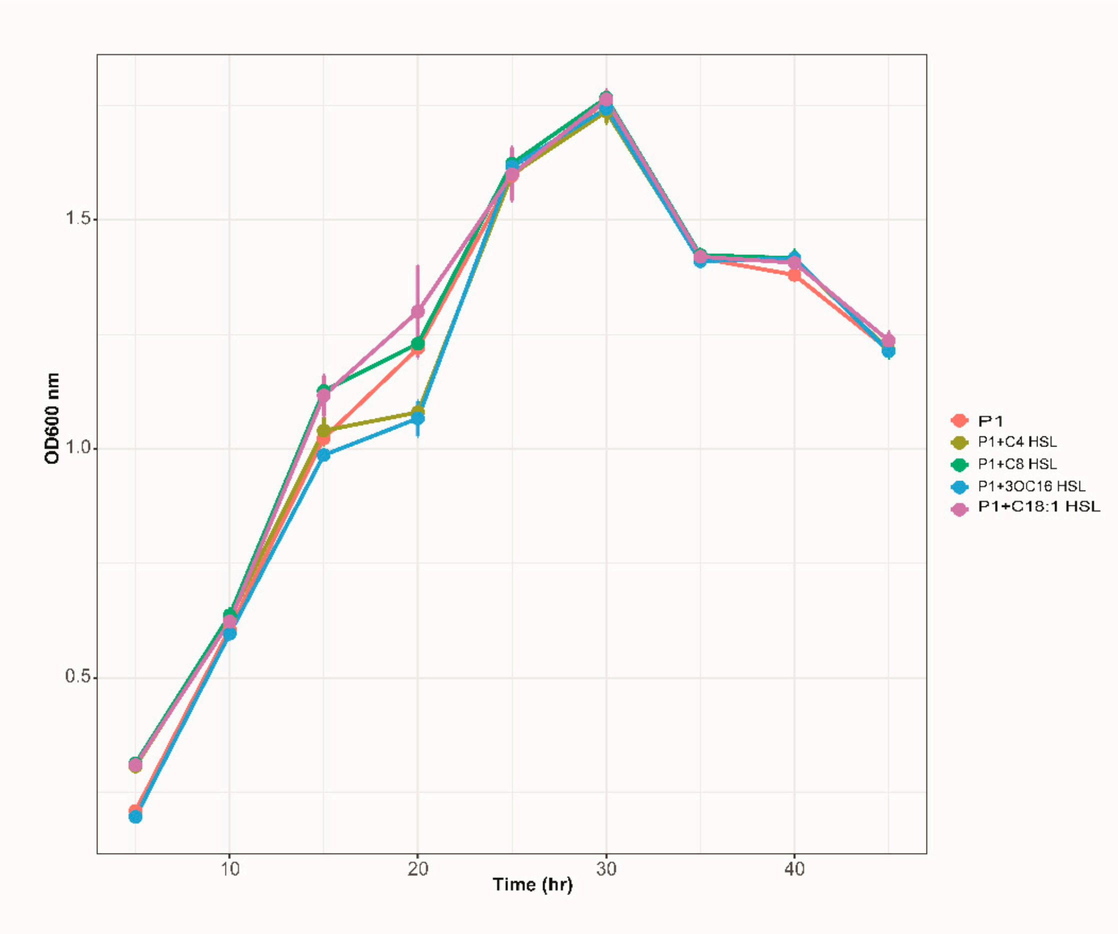

Figure S1. *P. galatheae* growth in the presence of different HSLs shows no significant increase compared to growth in the absence of HSL.

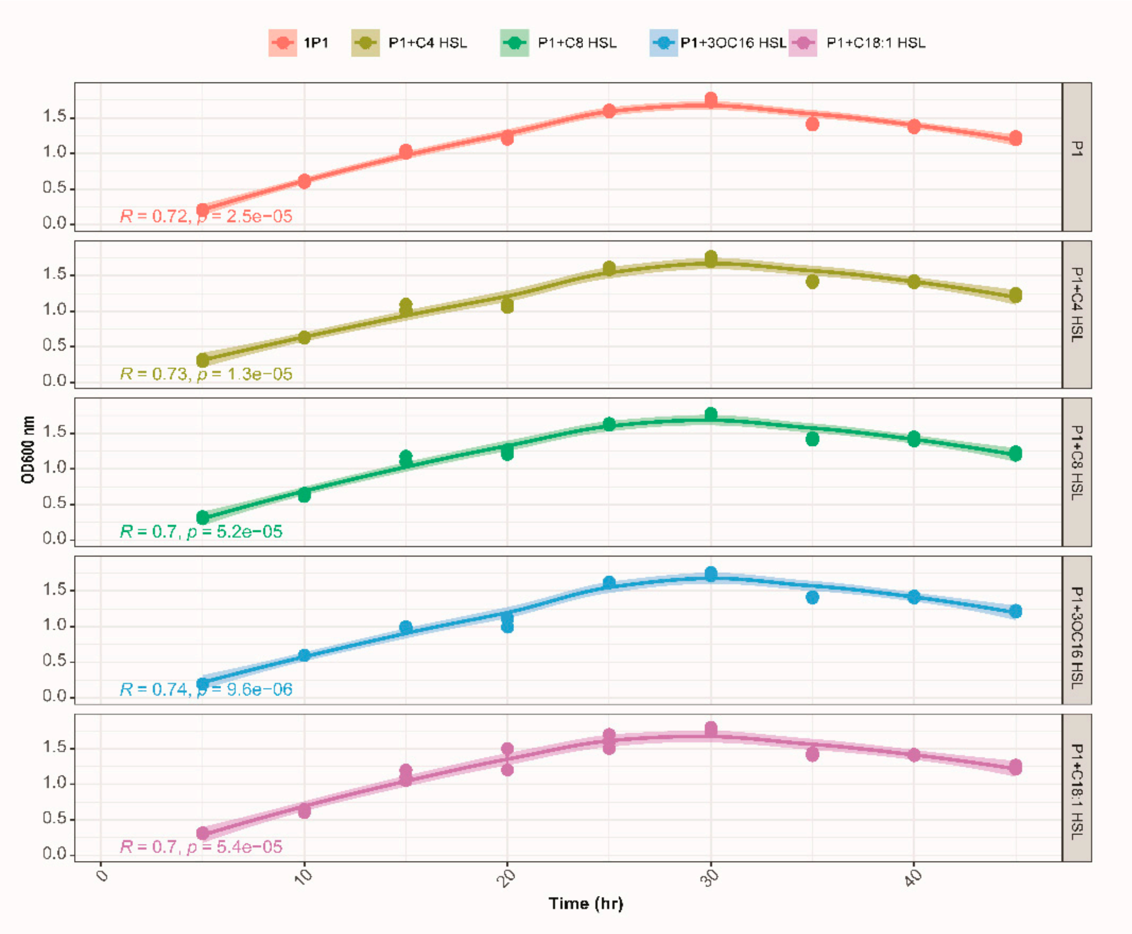

Figure S2. *P. galatheae* demonstrate no correlation in the presence of different

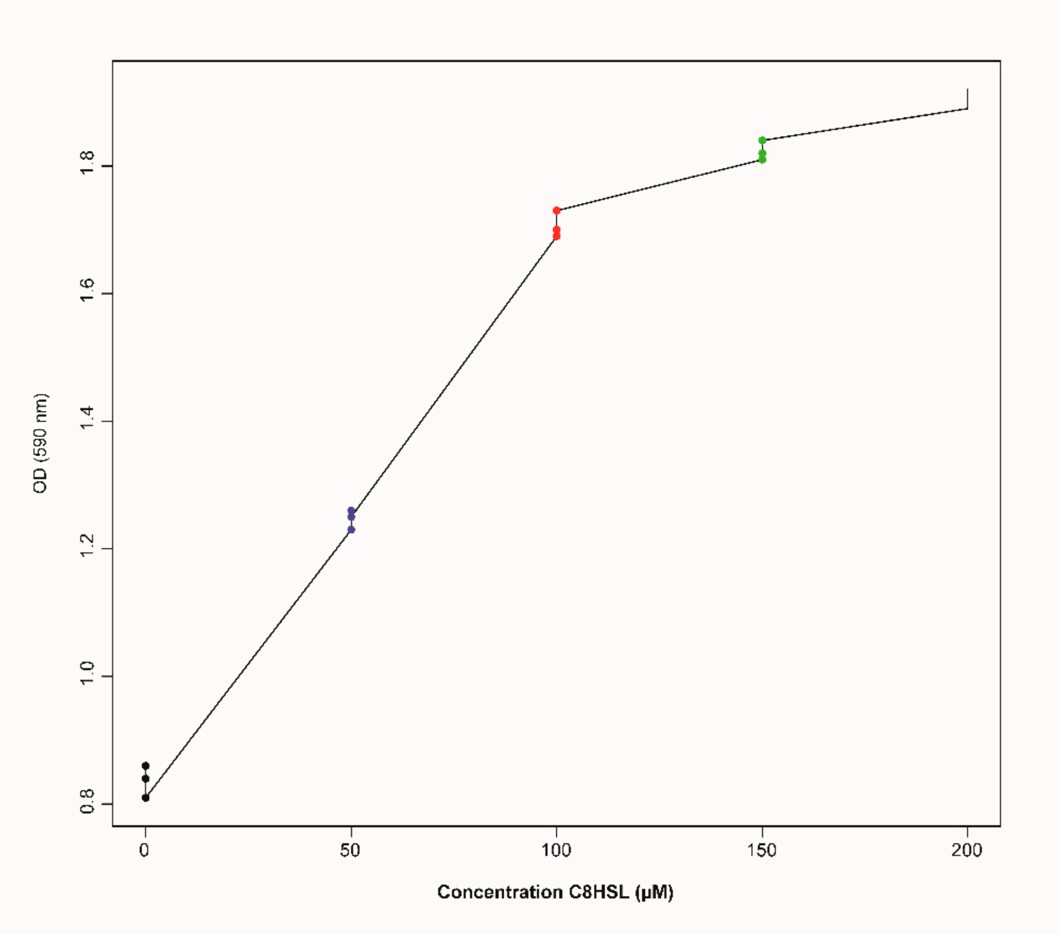

Figure S3. *P. galatheae* exhibits a positive correlation between biofilm formation and increasing concentration of C8 HSL
